# Supplementary material for: Development and Efficacy Evaluation of a Novel Nano-Emulsion Adjuvant for a Foot-and-Mouth Disease Virus-like Particles Vaccine Based on Squalane
Source: Nanomaterials (Basel). 2022 Nov 8;12(22):3934. doi: 10.3390/nano12223934 (PMC9698784; doi:10.3390/nano12223934)
Supplement: Supplementary file 1 [file nanomaterials-12-03934-s001.zip › nanomaterials-1947579-supplementary.pdf]

Supplementary Materials

Development and Efficacy Evaluation of a Novel Nano-Emulsion Adjuvant for a Foot-and-Mouth Disease Virus-like Particles Vaccine Based on Squalane

Tween60 were purchased from Sigma-Aldrich. Span85 were purchased from Aladdin. PEG-400 and squalene were purchased from Acmec. All other chemicals were of A.R. grade and were used as received without further purification.

Selection of Span85 and Tween60 ratios

Polyethylene glycol-400 was at quality of 0.8 g and 1.2g of the Span85 and Twenn60 was used. In the reaction system, the surfactant and co-surfactant were blended with the oil phase at a ratio of 1:9 to 9:1. In the surfactant, Span85 and Tween 60 in the ratio of 1:3, 1:2, 1:1, 2:1, 3:1. the appropriate ratio of Span85 to Tween60 was chosen by recording the maximum area of the pseudo-ternary phase diagram.

Selection of surfactant and co-surfactant ratios（Km）

Under the conditions, the ratio of Span85 to Tween60 was determined to be 2:1. In the reaction system, the surfactant and co-surfactant were blended with the oil phase at a ratio of 1:9 to 9:1. In the surfactant (Span85 and Twenn60) and co-surfactant (Polyethylene glycol-400), surfactant and anhydrous ethanol in the ratio of 1:1, 1:2, 2:1. The optimum ratio of surfactant and Polyethylene glycol-400 was selected by recording the maximum area of the pseudo-ternary phase diagram.

Expression, purification, and assembly of the FMDV capsid protein

The FMDV capsid proteins (His-Sumo-VP0, His-Sumo-VP1, and His-Sumo-VP3) were obtained using an Escherichia coli expression system that has been established by our laboratory, and the purification of structural proteins and assembly of VLP were performed according to previously described methods.30 The His-SUMO label was added to the structural protein of FMDV. Then, the structural protein of FMDV was purified by Ni2+-resin. From the purified proteins (His-SUMO-VP0, His-SUMO-VP1, and His-SUMO-VP3), the His-Sumo moieties were removed by SUMO protease, and the proteins were identified by sodium dodecyl sulfate polyacrylamide gel electrophoresis (SDS-PAGE) and western blot. The digested proteins were assembled into VLP in assembly buffer. The assembled particle size was measured by dynamic light scattering (DLS) with a Zetasizer-Nano (Malvern Zetasizer Nano ZS90; Worcestershire, UK). The particles of VLP were separated from the assembled sample using a protein purifier (AKTA pure 25; GE, NY, USA). The morphology of the particles was observed by transmission electron microscopy (TEM) (HT7700; Hitachi, Tokyo, Japan) after dyeing with phosphotung-stic acid.
